# Functions of Snake Sloughs in Bird Nests Vary with Habitats: A Test of the Anti-Predation Hypothesis

**DOI:** 10.3390/ani13081337

**Published:** 2023-04-13

**Authors:** Jinmei Liu, Laikun Ma, Yameng Jin, Fangfang Zhang, Xintong Li, Wei Liang

**Affiliations:** 1 Ministry of Education Key Laboratory for Ecology of Tropical Islands, Key Laboratory of Tropical Animal and Plant Ecology of Hainan Province, College of Life Sciences, Hainan Normal University, Haikou 571158, China; 2 School of Life Sciences, Hebei University, Baoding 071002, China; 3 Department of Biology and Food Science, Hebei Normal University for Nationalities, Chengde 067000, China

**Keywords:** anti-predator strategy, habitat, nestbox, nesting material, snake slough

## Abstract

**Simple Summary:**

The anti-predatory effect of snake sloughs in bird nests may vary with different types of habitats. This study showed that snake sloughs in bird nests at one study site reduced the predation rate, whereas no such effect was observed at two study areas, suggesting that the anti-predation function of snake sloughs in bird nests is associated with the major nest predators present in different habitats.

**Abstract:**

Snake sloughs in bird nests can reduce nest predation and serve as an anti-predator strategy. However, the anti-predator function of snake sloughs in nests has only been tested twice, and it is difficult to speculate around the origin of the differences, which may well include habitat, as predator species and predation risk vary in different habitat types. Habitat would be a good place to speculate as to how differences in habitats could explain differences in responses by nest predators. Thus, we selected three different habitats, namely, the Diaoluoshan National Nature Reserve in Hainan (DLS, tropical forest), the Hainan Normal University campus (HNU, urban area), and Qingchuifeng National Forest Park in Hebei (QCF, suburban area), to verify the anti-predator function of snake sloughs in bird nests. The experimental results showed that snake sloughs in the nests reduced the predation rate of the experimental nests in HNU, whereas no such effect occurred in DLS and QCF. This suggests that the anti-predatory function of snake sloughs may not be the same over some environmental gradients and could be dependent on the species of nest predators and food resources in the habitat, which does not apply to all types of habitats.

## 1. Introduction

Birds (except brood parasitic birds) use a wide variety of plant, animal, or artificial materials to construct nests to provide suitable sites for offspring survival and development [1,2]. The nest structure and nesting materials have important implications for the breeding behavior and reproductive success of birds [3,4]. In particular, the selection of suitable nesting material can provide specific benefits for both the parents and offspring, such as reduced embryonic heat loss [5] and parental energy consumption [6], reduced numbers of ectoparasites [7,8], improved pairing success [9,10], and reduced nest predation rates [11,12].

In studies of nesting materials, the antimicrobial and antiparasitic functions of green plants [8,13,14,15] and that of thermal insulation of feathers [16,17,18] have received considerable attention in the field of evolutionary ecology [2,4]. Snake sloughs are common nesting materials for some hole-nesting bird species [19,20,21,22,23,24,25,26,27]. However, few studies have explored the function of snake sloughs as nesting materials in bird nests. Previously, researchers speculated that birds add snake sloughs to their nests as an anti-predator strategy, i.e., the function of snake sloughs was to scare off nest predators and reduce predation rates [19]. However, this hypothesis was not verified until 2006 when Medlin and Risch found that nests of the great crested flycatcher (*Myiarchus crinitus*) with snake sloughs were effective in deterring mammalian predation, especially from the southern flying squirrel (*Glaucomys volans*); thus, flycatchers and other species may have evolved the behavior of including snake sloughs as nesting material to deter predation [22]. However, experiments by Alfréd and Prokop (2011) found that the majority of female great reed warblers (*Acrocephalus arundinaceus*) that were tested in the early stages of nest building immediately detected and incorporated experimental snake sloughs into the nest structures, but snake sloughs in nests of great reed warblers failed to reduce nest predation rates. They suggest that snake sloughs in great reed warbler nests may serve as a post-pairing signal revealing female parental quality [24]. Liu and Liang (2021b) observed snake sloughs in nests of crested mynas (*Acridotheres cristatellus*) in different stages of breeding [27]; later, Liu and Liang (2021a) verified the anti-predator function of snake sloughs and found that the addition of snake sloughs to nests was effective in deterring predation by the maritime striped squirrel (*Tamiops maritimus*), and by manipulating visual or olfactory cues, results showed that predators were deterred by the visual cues of the snake sloughs in the nests [12].

The anti-predator function of snake sloughs in nests was only tested twice, and it is difficult to speculate as the origin of the differences, which may well include habitat. Rather, one would expect different adaptations depending on the structure of the snake slough in different types of habitats, as predator species and predation risk are different. We think it would be a good place here to speculate as to how differences in habitats could explain differences in responses by nest predators. Differences in habitat conditions can affect the breeding behavior of birds. For example, populations of great tits (*Parus major*) residing in four diverse habitats of Pina (maritimes *Pinus pinaster*, scots pines *Pinus sylvestris*, pyreneans *Quercus pyrenaica*, zeen oaks *Quercus faginea*), Mariola (aleppo pines *Pinus halepensis*), Font Roja (holm oaks *Quercus ilex*), and Sagunto (oranges *Citrus aurantium*) were able to adjust the nest size and nest composition to local conditions, with significant differences in nest quality and nest composition between habitats [28]. In addition, differences in geographical variations can also affect the breeding behavior of birds. A typical example is the latitudinal variation in clutch size, where birds lay more eggs at higher latitudes than in the tropics [29]. On the one hand, clutch size may be limited by food resources; on the other hand, it is affected by the risk of predation [30,31]. Generally speaking, birds in tropical regions have higher nest predation risk and lower food availability than birds in temperate regions during the breeding season [30,32,33].

In this study, we conducted experiments with the following three objectives: (1) to test the reproducibility of previous experiments at the same site, Hainan Normal University (thereafter HNU), on the basis of the results of Liu and Liang (2021a); (2) to investigate the anti-predator function of snake sloughs in nests under different habitat conditions through selecting a tropical rainforest in Diaoluoshan (DLS), Hainan, which is of similar latitude to HNU but in a completely different habitat; (3) to test the effect of geographical differences on the anti-predator function of snake sloughs through an experiment in Qingchuifeng (QCF) National Forest Park in Hebei, China, which has a large latitudinal difference from Hainan.

## 2. Materials and Methods

### 2.1. Study Area

**Figure 1:**
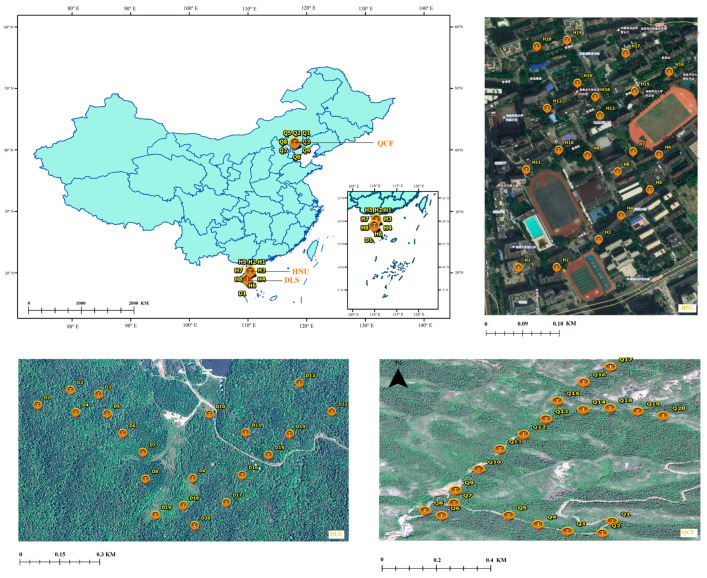
The study area (Q1–Q20 indicates the nest box sample site of QCF; H1–H20 indicates the nest box sample site of HNU; D1–D20 indicates the nest box sample site of DLS).

The Longkun South Campus of HNU (19°59′ N, 110°20′ E, 10 m in elevation) is located in Haikou, Hainan Province, China, covering an area of approximately 26 ha (Figure 1). The main vegetation species on the campus include coconuts (*Cocos nucifera*), royal palms (*Roystonea regia*), orchid trees (*Bauhinia blakeana*), and herba ficus (*Ficus microcarpa*) [34]. Hole-nesting birds are the oriental magpie-robin (*Copsychus saularis*) and crested myna (*Acridotheres cristatellus*) (see Liu et al. 2020 for further details [35]), and the major predator of birds on the campus is the maritime striped squirrel [12].

DLS (18°43′–58′ N, 109°43′–110°03′ E, with a total area of approximately 37,900 ha and 800 m in elevation at experimental site) is located in the southeastern part of Hainan Island, China (Figure 1). The altitude ranges from 50 to 1499 m, with abundant rainfall and an annual rainfall of 1870 to 2760 mm. The park is rich in plant resources, diverse types of vegetation, and complex floristic composition, and it has a wide range of animal and bird predator species [36]. The forest coverage rate reached 98.8%, with the tropical virgin forest area accounting for about one-third of the forest area [37]. The nest predators in the park are snakes, birds, the maritime striped squirrel, Pallas’s squirrel (*Callosciurus erythraeus*), and the northern tree shrew (*Tupaia belangeri*) [37].

QCF (40°57′–41°01′ N, 117°56′–118°01′ E) located in the suburbs Chengde, Hebei, north China, has a total area of 10,600 ha (Figure 1). The altitude ranges from 313 to 1074 m, with an average annual temperature of 8.9 °C and an average annual precipitation of 587 mm [38]. The park is home to a wide variety of plant species, including the oriental arborvitae (*Platycladus orientalis*), dahurian larch (*Larix gmelinii*), and false acacia (*Robinia pseudoacacia*). The nest predators in the park are snakes and chipmunks (*Tamias sibiricus*) (Ma L. 2020–2022, unpublished data).

### 2.2. Study Species

**Figure 2:**
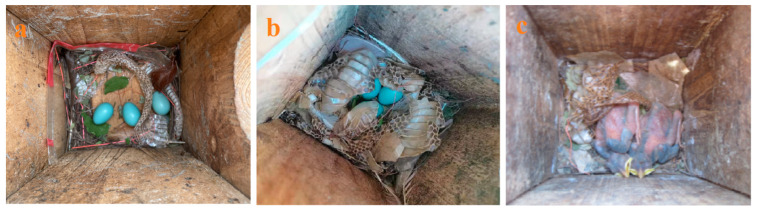
Snake sloughs in nests of crested mynas during the breeding season ((**a**) the egg laying stage; (**b**) the incubation stage; (**c**) the nestling stage).

The crested myna is a widely distributed species in south China as well as India [39,40]. The crested myna subspecies *A. c*. *brevipennis* [40] is a common local resident with a large population on tropical Hainan. Its breeding period is from March to August, nesting in tree holes or building holes [41], or nest boxes [12]. The addition of snake sloughs occurs in different stages of breeding (Figure 2), with a proportion of 38.9% being during the nest-building period [42]. Snake slough in myna nests does not promote nestling growth [42] but can effectively reduce the risk of nest predation [12]. Maritime striped squirrels are the nest predators of crested mynas [27].

### 2.3. Data Collection

**Figure 3:**
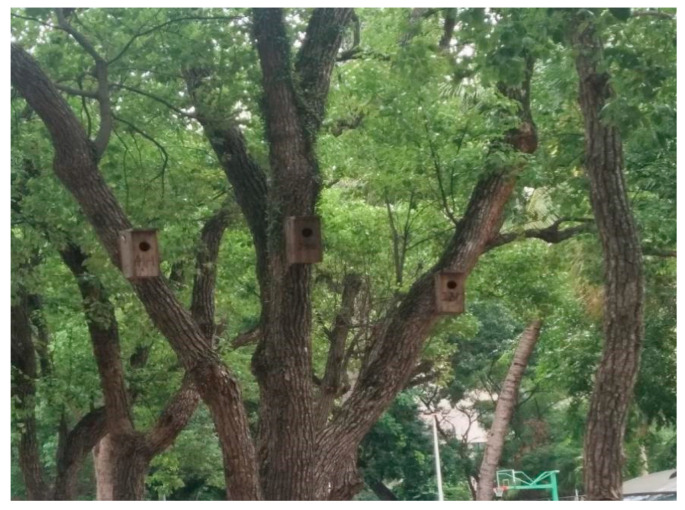
Three nest boxes were suspended on tree trunks in one nest box sample site.

In this experiment, wooden nest boxes (L × W × H = 15 × 15 × 30 cm; box opening diameter 6 cm, *n* = 180) used by Liu and Liang (2021b) [27] were suspended 3 m above the ground on tree trunks during breeding season in HNU (7–18 August 2020), DLS (14–25 May 2020), and QCF (1–12 August 2020), as shown in Figure 3. Every three nest boxes were considered as one nest box sample site, and the distance between the nests within a nest box sample site was maintained within 5 m to ensure three nest boxes within a nest box sample site have similar chances of being found by predators; the distance between nest box sample sites was greater than 60 m to ensure that the nest boxes were located independently from each other.

**Figure 4:**
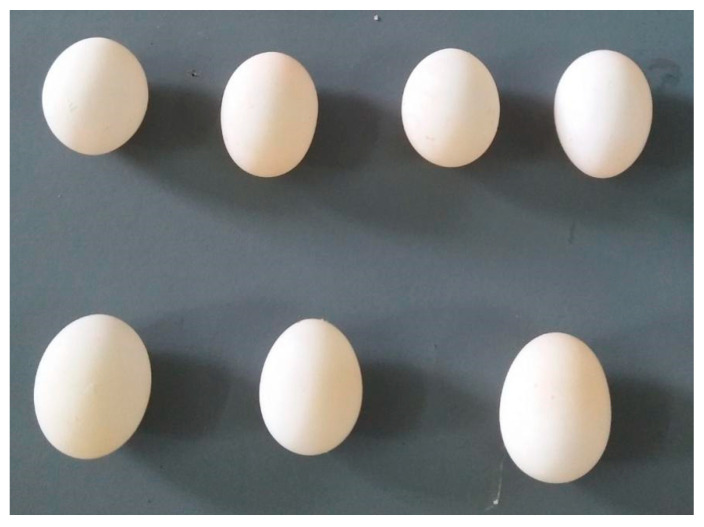
Eggs of the white-rumped munia (*Lonchura striata*) used in this study.

Cup nests weaved from hay and two captive-bred white-rumped munia (*Lonchura striata*) eggs (1.01 ± 0.15 g in egg mass, 15.20 ± 0.64 mm in egg length, 11.93 ± 0.68 mm in egg width; *n* = 20) were placed in all nest boxes, as shown in Figure 4. All munia eggs used in the experiment were all unfertilized eggs and were purchased online (Taobao Inc., Hangzhou, China). A total of 180 nest boxes were suspended, with each of 60 boxes at HNU, DLS, and QCF. The snake sloughs used in the experiment (Chinese cobra, *Naja atra*) were obtained from crested myna’s nests in a local farm [12].

Three nest boxes of a nest box sample site were randomly placed separately in DLS and QCF: a brownish gray cloth (10 cm) was added to the nest boxes (the experimental group EC), 10 cm of snake sloughs was added to the nest boxes (the experimental group E), and no treatment was applied to the nest boxes (the control group C). Three treatments were randomly placed correspondingly in three nest boxes of a nest box sample site in HNU: 10 cm of snake slough was added to the nest boxes (the experimental group E), 10 cm of snake sloughs was suspended at the mouth of the nest boxes (the experimental group ES), and no treatment was applied to the nest boxes (the control group C). As the experimental results of Liu and Liang (2021a) found that there was no significant difference in predation rate between the group with cloth boxes added and the group without treatment [12], HNU boxes therefore did not have the brown cloth in the nests. The experimental group ES in HNU was added to examine visual cues for snake sloughs.

To determine the type of predator, 12 nests at each site were recorded using a WIFI/P2P miniature network camera (HD99S-32G, Shenzhen Skywork Digital Co. Ltd., Shenzhen, China) with a portable battery (ROMOSS, Shenzhen Romoss Technology Co. Ltd., Shenzhen, China) as a continuous power supply [12].

The period of this experiment was set to 12 days, which is similar to the period of time for most incubation periods of hole-nesting birds in the study area. All nest boxes were monitored manually to record nest predation in the nests on the afternoon of the third, sixth, and ninth days of the experiment. Eggs were considered to have been predated if the white-rumped munia eggs showed bite marks or if the eggs disappeared during the experimental cycle [12].

### 2.4. Data Analysis

To analyze the effect of treatments on nest predation (whether the eggs of every nest box had been preyed after 12 days) at different regions, because the data is binary, we applied generalized linear mixed models (GLMM) with binomial distribution using the function glmer in R package lmer between three sites [43]. The treatments (C, E, ES, EC) and regions (HNU, DLS, QCF) were set as fixed effects, and the nest box sample site was considered as a random effect. Then, we used the same method to compare nest predation rates between different treatments within every site and only set treatments as fixed effects. We fitted the survival curves at three regions by survival analysis in Kaplan–Meier estimation using the function survfit in R package survival [44]. All data analyses for this study were performed in R v.4.2.0 (R CoreTeam, 2022).

## 3. Results

**Figure 5:**
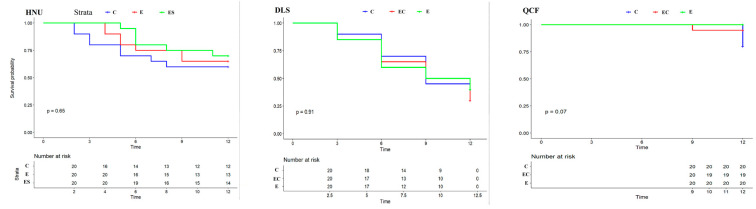
Egg survival curves of different treatments in three regions (*p* refers to the significance of survival curves in different treatments).

Egg survival curves showed that nest predation events in HNU and DLS occurred earlier than QCF, and the probability of nest predation events in HNU and DLS was higher than that in QCF. Conversely, there was no significant difference in the egg survival curve among different treatments in HNU, DLS, and QCF (*p* = 0.65, *p* = 0.91, *p* = 0.07, respectively; Figure 5). The nest predation rate in DLS was significantly different from HNU and QCF (*p* = 0.045, *p* < 0.001, respectively; Table 1), and the results showed a significant difference of the nest predation rate compared with HNU (*p* = 0.045 for DLS and *p* < 0.014 for QCF).

Through the video recordings, we found that the nest predators in HNU were maritime striped squirrels and that nest predation rates differed significantly between treatment groups in the nest boxes. The experimental groups E and ES exhibited significant and highly significant lower nest predation rates than that of the control group C (*p =* 0.010 and *p* = 0.003, respectively; GLMM) (Table 2). There was a significant difference between the E and ES experimental groups (*p* = 0.016; GLMM).

There was a wide variety of nest predators in DLS, including flying squirrels (*Hylopetes* spp.), squirrels (*Callosciurus* spp.), and birds. The nest predators in QCF were the chipmunk and maritime striped squirrel. The nest predation rates did not differ significantly (*p* > 0.05) between any of the nest box treatment groups in DLS and QCF (Table 2).

## 4. Discussion

Our results showed that survival curves of different treatment groups were not different in the three regions. However, DLS and HNU predation events occurred earlier than QCF, which may have been due to different predation pressures and food resources. The first model, the nest predation rate in DLS, was significantly higher than that in HNU and QCF, and the nest predation rate in HNU was significantly higher than that in QCF, which may have been due to differences in predator species and predation risk. The lack of significance (although in the case of treatment E, the *p*-value was very close to the statistically significant threshold) highlighted that there were no differences between treatments when controlling for geographic regions (Table 1). In HNU, the treatment E and ES were significantly lower than that of the treatment C. This indicated that snake sloughs in the nests in HNU could reduce nest predation rates. The result was similar to the results previously reported by Liu and Liang (2021a). In DLS and QCF, nest predation rates were not different between the experimental groups (EC, E) and the control group (C), which did not support the anti-predation function of snake sloughs. Our results suggested that the anti-predatory function of snake sloughs may not be the same over some environmental gradients, potentially being dependent on the species of nest predators and food resources in the habitat.

Our research results showed that, compared with QCF, nest predation events of DLS and HNU occurred earlier and higher, which was understandable. Previous studies have found a trend of high predation rates among terrestrial and marine invertebrates, bird nests, and insects at low latitudes [45,46], which may have been due to high biodiversity [47] and high risk of predation in tropical areas [32]. Our results were consistent with this trend. In addition, our results showed that egg predation events in QCF occurred on days 9–12, which may have been related to the availability of food. The nest predators in QCF were the omnivorous chipmunks and maritime striped squirrels (Ma L. 2020–2022 unpublished data), with an abundance of food resources (such as pinecones). Therefore, the chipmunks and squirrels probably had relatively little difficulty obtaining food and did not venture into the nest boxes to hunt the eggs in the early stages of the experiment.

The effectiveness of the anti-predatory strategy of snake sloughs in nest boxes differed among the three areas, which we believe was mainly owing to the differences in habitat types and the nest predator types in the three areas. Liu and Liang (2021a) found that snake sloughs in bird nests were able to deter bird predators, such as the maritime striped squirrel [12]. Similarly, in this study, snake sloughs from crested myna nests were found to significantly deter the squirrels and reduce nest predation rates during the breeding season in HNU, which confirmed earlier findings from Liu and Liang (2021a). The DLS is part of a tropical forest, which is the most species-rich and structurally complex forest ecosystem type on land [36]; our results showed that snake sloughs in the nest did not reduce nest predation. The DLS has a wide variety of bird nest predators, such as snakes, birds, squirrels, and tree shrews [37], and the nest predators found in the current study were flying squirrels and some ants and bird predators. Compared with HNU, which have complex and different nest predator species, this difference may be one of the reasons for the difference in effectiveness of the anti-predatory strategy of snake sloughs in the three areas. In addition, we suggested that the ease of access to food by nest predators also affects the nest predation rates. Predators in HNU were the omnivorous maritime striped squirrels, with an abundance of food resources in the study area. There are dwellings and canteens in HNU, with food scraps such as rice and noodles being plentiful. In contrast, food is highly attractive in tropical forest environments; hence, predators are more inclined to take high risks when faced with food, and thus, avian predators in DLS are more likely to take risks when faced with snake sloughs in bird nests. In QCF, the nest predators were chipmunks and maritime striped squirrels, and the nest predation rate of QCF was significantly lower than that of DLS and HNU; nonetheless, snake sloughs in the nest also did not reduce nest predation, which may have been related to the density of nest predators, especially maritime striped squirrels.

There are some limitations to our experiment. For example, owing to the limited number of cameras, only a few nests were videotaped, and the identification of avian predators on the basis of bite marks was possible only in a portion of the nests. Although there were many species of avian predators in DLS, the identification of multiple avian predators was unclear, and future experiments need to be conducted to clearly identify the species of nest predators of cavity-nesting birds in DLS. In addition, we will conduct similar experiments in the future, such as the effect of snake sloughs in reducing the number of parasites in the nest and the effect of the number of snake sloughs on the parasites.

## 5. Conclusions

In this study, the anti-predator function of snake sloughs from crested mynas’ nests was verified at three sites with different habitats. In HNU, snake sloughs in crested mynas’ nests were able to reduce nest predation rates, and the results were similar to those previously reported by Liu and Liang (2021a). In DLS and QCF, snake sloughs did not reduce nest predation. The results suggested that the anti-predatory function of snake sloughs may be related to habitat features and geographic gradients. The experimental results in DLS differed from those in HNU, because DLS is part of a species-rich tropical forest with a complex forest structure and diversity of potential predator species for birds, which may lead to the ineffectiveness of snake sloughs in nests to reduce nest predation rates in this area. Adding snake sloughs to bird nests is an adaptive behavior. As far as we know, crested mynas do not choose to build nests in tropical rainforests. Of course, snake sloughs from crested mynas’ nests may have other functions, and more research is needed on other functions of snake sloughs in bird nests.

## Figures and Tables

**Table 1:** Comparison of nest predation among different treatment groups and regions by GLMM (the treatment reference level was the control group C; the region reference level was DLS).

Fixed Effects	Estimate	Std. Error	z-Value	*p*-Value
Treatments				
Intercept	1.831	0.843	2.173	0.030
EC (egg and cloth inside)	−0.596	0.703	−0.847	0.397
E (egg and slough inside)	−1.214	0.628	−1.934	0.053
ES (egg and slough outside)	−1.362	0.99	−1.376	0.169
Regions				
HNU	−2.415	1.206	−2.003	0.045
QCF	−5.512	1.437	−3.836	<0.001

**Table 2:** Comparison of nest predation among different treatment groups at three regions by GLMM (the reference level was the control group C).

Treatment	Estimate	Std. Error	z-Value	*p*-Value
HNU				
Intercept	−13.558	3.917	−3.462	<0.001
E (egg and slough inside)	−15.029	5.858	−2.566	0.010
ES (egg and slough outside)	−29.213	10.005	−2.920	0.003
DLS				
Intercept	0.931	0.546	1.704	0.088
EC (egg and cloth inside)	−8.254 × 10^−6^	0.718	0	1
E (egg and slough inside)	−0.484	0.702	−0.690	0.490
QCF				
Intercept	−12.620	35.530	−0.355	0.722
EC (egg and cloth inside)	−15.710	60.180	−0.261	0.794
E (egg and slough inside)	−1279	1.501 × 10^7^	0	1

## Data Availability

The datasets used in this study are available from the corresponding author upon reasonable request.
